# Evaluation of preanalytical factors on quantification of amyloid-β (1-42), phosphorylated tau 181, and total tau in cerebrospinal fluid

**DOI:** 10.1093/labmed/lmaf044

**Published:** 2025-08-21

**Authors:** Tiffany R Allison, Sonia L La’ulu, Kelly Doyle, Heather A Nelson

**Affiliations:** ARUP Institute for Clinical and Experimental Pathology, Salt Lake City, UT, United States; ARUP Institute for Clinical and Experimental Pathology, Salt Lake City, UT, United States; ARUP Institute for Clinical and Experimental Pathology, Salt Lake City, UT, United States; Department of Pathology, University of Utah School of Medicine, Salt Lake City, UT, United States; ARUP Institute for Clinical and Experimental Pathology, Salt Lake City, UT, United States; Department of Pathology, University of Utah School of Medicine, Salt Lake City, UT, United States

**Keywords:** Alzheimer disease, CSF biomarkers, preanalytical, amyloid beta, tau

## Abstract

**Introduction:**

Cerebrospinal fluid (CSF) biomarkers for Alzheimer disease are US Food and Drug Administration approved and implemented on automated platforms, allowing for widespread use and higher throughput. Although low-bind polypropylene tubes for accurate quantification of amyloid-β (Aβ) have been well studied, less is known about the impact of other common preanalytical variables on quantification of biomarkers for Alzheimer disease. This study evaluated the effects of refrigerated transportation and hemolysis on the concentrations of Aβ42, phosphorylated tau 181, and total tau.

**Methods:**

The Roche Diagnostics Elecsys β-Amyloid (1-42) CSF II, Elecsys Phospho-Tau (181P) CSF, and Elecsys Total-Tau CSF assays were used on the Roche cobas pro e 801 platform to measure protein concentrations in residual CSF samples. Paired-difference testing was performed to determine the effects of simulated transportation and hemolysis on each analyte.

**Results:**

For all 3 analytes, less than 10% difference was observed between the concentrations measured on day 0 and after 14 days of transportation and refrigeration. In contrast, 2.26 g/L free hemoglobin resulted in more than 10% negative bias in Aβ42 measurement compared with the 0 g/L control but did not affect phosphorylated tau 181 or total tau concentrations.

**Discussion:**

Refrigerated transportation did not affect the analysis of Aβ42, phosphorylated tau 181, or total tau, whereas hemolysis can negatively affect results of Aβ42.

## Introduction

Alzheimer disease is the most common cause of dementia, affecting an estimated 6.9 million Americans older than 65 years of age.^1^ The disease is characterized pathologically by accumulation of amyloid-β (Aβ) plaques and intraneuronal neurofibrillary tangles composed of hyperphosphorylated tau protein (p-Tau).^2^ Amyloid-β peptides are formed from cleavage of the transmembrane amyloid precursor protein. Due to its hydrophobic elements, Aβ tends to aggregate, which occurs in a concentration-dependent manner. The most abundant form of Aβ found in extracellular plaques is a 42-amino acid peptide, Aβ42.^3^ As Aβ42 aggregates into fibrils and plaques in the brain, less of the peptide can diffuse into the cerebrospinal fluid (CSF), resulting in decreased CSF Aβ42 concentrations in individuals with Alzheimer disease. Tau is a protein involved in microtubule stabilization; it occurs in 6 isoforms based on alternative splicing and has several phosphorylation sites. In Alzheimer disease, tau is hyperphosphorylated, detaches from the microtubules, and accumulates in cell bodies and dendrites, forming neurofibrillary tangles.^2^

Because of their role in the pathogenesis of Alzheimer disease, these proteins have become important biomarkers for its diagnosis. When measured in CSF, a combination of decreased concentrations of Aβ42 and increased concentrations of p-Tau and total tau (t-Tau) are consistent with Alzheimer disease.^3^ Cerebrospinal fluid biomarkers offer greater accessibility and lower costs than amyloid positron emission tomography and may play a pivotal role in qualifying patients for new US Food and Drug Administration–approved treatments that slow disease progression.

Multiple immunoassays are available to measure Aβ, p-Tau, and t-Tau in CSF; however, their accuracy may be limited by preanalytical factors if not well controlled. Preanalytical factors that can affect the concentration of these proteins in CSF include sample collection, handling, storage, tube type, and transportation.^4-6^ Aβ42 is especially sensitive to preanalytical variations; in particular it is susceptible to adhering to plastic surfaces.^4,7-9^ Therefore, preanalytical protocols were established to standardize handling of CSF specimens to minimize the impact of preanalytical factors on measurement of Aβ and tau proteins.^4,10^

The Roche Diagnostics Elecsys β-Amyloid (1-42) CSF, Phospho-Tau (181P) CSF, and Total-Tau CSF immunoassays are available on a fully automated platform, allowing for robust, high-throughput measurement of Aβ42, tau phosphorylated at threonine 181 (p-Tau 181) and t-Tau. Because the CSF ratios demonstrate superior diagnostic performance compared with the individual analytes in diagnosing Alzheimer disease, the results are reported as 2 ratios: p-Tau 181/Aβ42 and t-Tau/Aβ42.^11-13^ Many of the recommendations published in the Alzheimer Association’s international guidelines for handling of CSF for routine clinical measurement of Aβ and tau were subsequently integrated into the manufacturer’s instructions for use^14-16^—notably, collecting directly into low-bind polypropylene tubes, limiting handling after collection (no centrifugation, mixing/inverting, or tube transfers), and storing at 2 °C to 8 °C for 14 days or at –15 °C to –25 °C for 8 weeks. Avoiding mixing/inversion of samples is challenging during transportation, especially if samples are transported by car or air to a different facility.

It was also indicated that samples should be visually inspected for hemolysis and not to use samples that appear reddish. The interference studies published by the manufacturer, however, indicated that there was no interference from hemolysis up to 150 mg/L (0.15 g/L) tested. This low concentration is not discernable by eye, raising concerns for how undetected low levels of hemolysis might affect results.

Therefore, the aims of this study were to (1) determine the impact of simulated refrigerated transportation and (2) evaluate the effect of hemolysis on the analytical results of Aβ42, p-Tau 181, and t-Tau measured in CSF using the Elecsys assays on the Roche cobas platform.

## Methods

### CSF samples

Residual deidentified CSF specimens were used for all studies. Residual specimens were obtained from frozen storage (–20 °C), thawed at room temperature for 30 minutes, and mixed upright, gently rolling each specimen 20 times. For some studies, samples were pooled, mixed, and aliquoted into low-bind polypropylene tubes. Protocols for using residual patient specimens were approved by the University of Utah Institutional Review Board (IRB No. 00007275).

### Immunoassays

Analyses were performed on a Roche cobas pro e 801 system using the Elecsys β-amyloid (1-42) CSF II, Elecsys Phospho-Tau (181P) CSF, and Elecsys Total-Tau CSF immunoassays.^14-16^ Each assay is a 2-step antibody sandwich electrochemiluminescence immunoassay. The analytical measurement ranges are 150 to 2500 pg/mL, 8.0 to 120 pg/mL, and 80 to 1300 pg/mL for Aβ42, p-Tau181, and t-Tau, respectively.

### Refrigerated transportation stability study

Residual CSF specimens were combined to form 3 different sample pools with a low (318 pg/mL), intermediate (538 pg/mL), and high (1728 pg/mL) concentration of Aβ42. Approximately 2 mL of each sample pool was aliquoted into individual 2.5-mL low-bind polypropylene tubes. Tubes were stored at 2 °C to 8°C in a box that was rotated and shaken 5 times per day. The rotation ensured that the CSF sample contacted the cap to simulate the inversion and mixing that may occur during transit. Samples were rotated only for the first 3 days of the experiment, encompassing expected transit times. After 3 days of rotation, the samples were left stationary up to 14 days to confirm the manufacturer’s stability at 14 days of refrigeration. For each sample, a single measurement of Aβ42, p-Tau181, and t-Tau was made on day 0 and at 1, 2, 3, and 14 days stored at 2 °C to 8°C. The percentage difference in concentration measured at each time point was calculated from the initial concentration measured on day 0.

### Hemolysis investigation

A hemolysate concentrate was prepared from purified red blood cells. Briefly, the red blood cells were separated from plasma, washed with saline, pelleted, and subjected to osmotic shock by addition of an equal volume of clinical laboratory reagent water. The cells were vortexed, frozen overnight, then thawed at ambient temperature. After thawing, the lysate was centrifuged for 30 minutes at 1972*g* to remove cell debris. The free hemoglobin concentration in the lysate was measured using a Beckman Coulter DxH hematology analyzer. The final concentration was 153 g/L. Hemolysate was added to 4 CSF samples (2 pooled samples and 2 individual samples) to obtain aliquots with increasing hemoglobin. The final hemoglobin concentrations were 0, 0.00, 0.22, 0.90, and 2.26 g/L. Isotonic saline was added to the control to compensate for volume increase and to correct for dilution. Aβ42, p-Tau181, or t-Tau were measured in duplicate, and the mean was used to determine the percentage difference between each hemolysate spiked sample and the 0 g/L hemoglobin control.

### Data analysis

Data analysis was performed in Microsoft Excel. Graphs were generated in GraphPad Prism 8 software (GraphPad by Dotmatics). For all studies, the total allowable error was defined as ±10% of the initial value, which was derived from a conservative adoption of the manufacturer’s package insert.

## Results

### Refrigerated sample stability study

**Figure 1.**
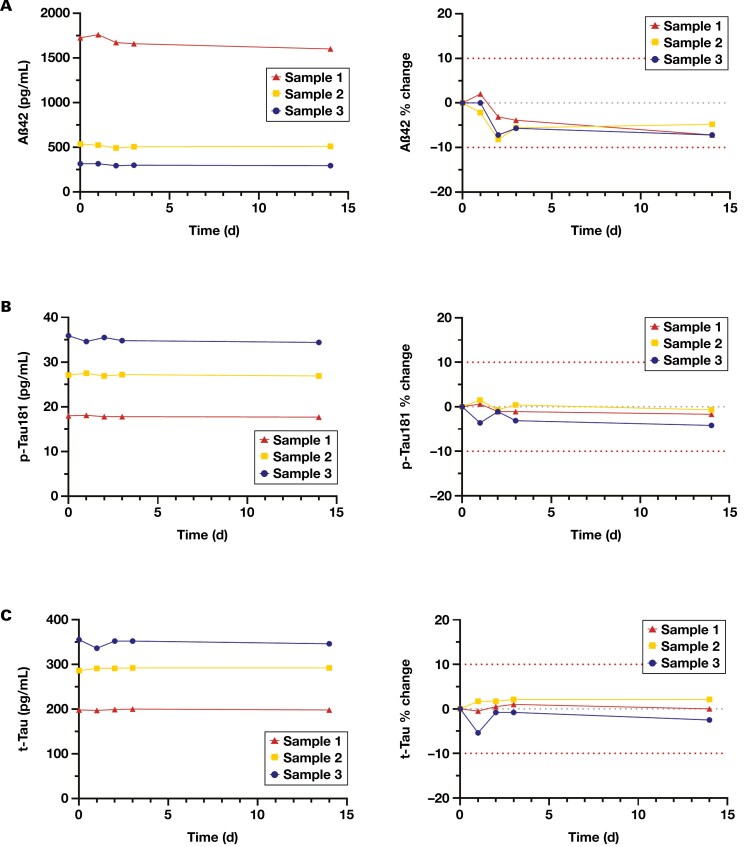
The effect of simulated refrigerated transportation (days 1-3) and storage (day 14) on concentration of (**A**) Aβ42, (**B**) p-Tau181, and (**C**) t-Tau in CSF. The left panel displays the results as a change in absolute concentration. The right panel illustrates the results as a percentage difference from the initial analyte concentration before transportation simulation (day 0). Each trace represents an individual sample. The outer red dotted lines represent ±10% difference in allowable error. The center gray dotted line represents 0% change. Aβ42 indicates 42-amino acid amyloid-β; CSF, cerebrospinal fluid; p-Tau181, tau protein phosphorylated at threonine 181; t-Tau, total tau protein.

The impact of simulated transportation and refrigerated storage on measurement of Aβ42, p-Tau181, and t-Tau are shown in Figure 1. All 3 analytes met our acceptance criteria (within ±10%) for the duration of the study. Aβ42 appeared to have a small but insignificant decrease in concentration by the second day that levelled off and did not decrease further (Figure 1A). At 14 days, there was less than an 8% decrease in Aβ42 concentration compared with day 0 for all 3 specimen pools. p-Tau181 and t-Tau were more robust to the transportation simulation, with observed relative differences of less than 5% for p-Tau181 and less than 3% for t-Tau at day 14 compared with day 0 (Figure 1B and 1C).

### Impact of hemolysis

**Figure 2.**
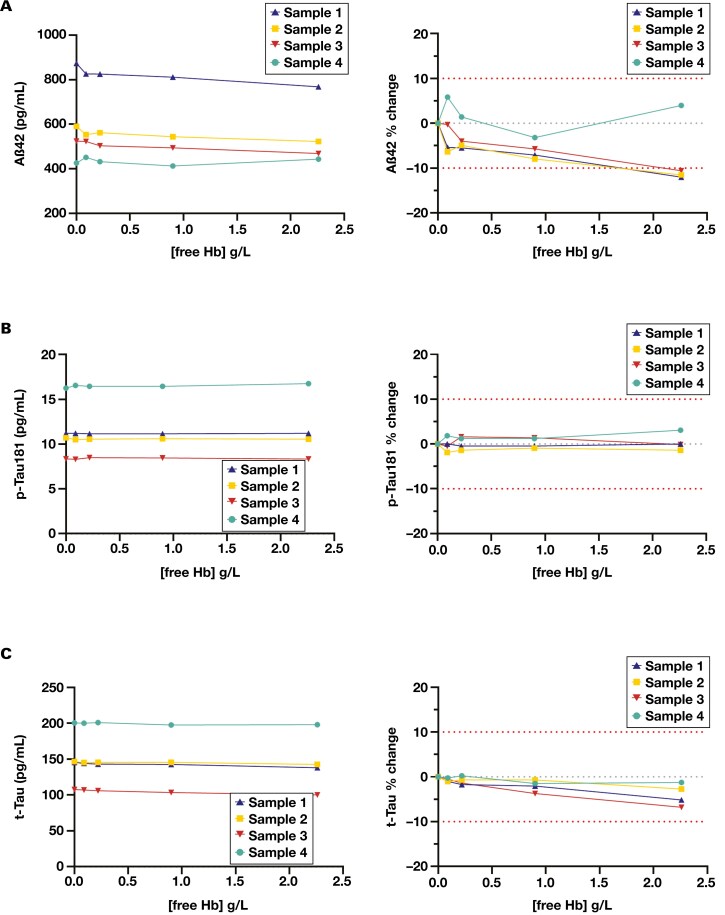
The effect of hemolysis on (**A**) Aβ42, (**B**) p-Tau181, and (**C**) t-Tau. The left panel displays the results as a change in absolute concentration. The right panel illustrates the results as a percentage difference from the initial analyte concentration (no hemolysate added). Each trace represents an individual sample. The outer red dotted lines represent ±10% difference in allowable error. The center gray dotted line represents 0% change. Aβ42 indicates 42-amino acid amyloid-β; Hb, hemoglobin; p-Tau181, tau protein phosphorylated at threonine 181; t-Tau, total tau protein.

The impact of increasing hemoglobin concentration on measurement of Aβ42, p-Tau181, and t-Tau are shown in Figure 2. For Aβ42, 3 of 4 samples had a more than 10% decrease in Aβ42 concentration in the 2.26-g/L hemoglobin sample compared with the nonhemolyzed control (Figure 2A). Each of the 3 samples exceeding 10% difference had an initial Aβ42 concentration greater than 480 pg/mL, failing our preset acceptance criteria. The only sample that was not affected by hemolysis was a low-concentration sample (427 pg/mL Aβ42). At 0.9 g/L or less hemoglobin, all hemolyzed samples had less than 10% difference in measured Aβ42 concentration.

For p-Tau181 and t-Tau, the relative difference in measured concentration between the nonhemolyzed control and the samples spiked with hemoglobin was less than 7% at all concentrations of hemoglobin tested (Figure 2B and 2C), which met our preset acceptance criteria of less than 10% change.

## Discussion

In this study, we evaluated the impact of simulated refrigerated transportation and hemolysis on Aβ42, p-Tau181, and t-Tau in CSF, measured on the Roche cobas platform. In adherence with the Alzheimer Association’s international guidelines for preanalytical handling of CSF, the manufacturer’s package insert included specific instructions for use pertaining to sample collection and handling. One directive was to avoid mixing or inverting samples, which stems from studies that have demonstrated the propensity of Aβ peptides to adsorb onto plastics.^6-8,17^ Although use of low-bind tubes can mitigate loss of Aβ on the plastic surface, there is risk of loss upon extended contact between the sample and the tube and concern for adsorption of the peptide to the plastic cap.^4,18^

Although avoiding mixing and inversion of samples is recommended, it is not always feasible to control for in daily practice, particularly if samples need to be transported externally to a different facility. While stored in shipping boxes and shuffled between vehicles or airplanes, the samples are tossed and turned. Freezing the samples for shipping is 1 way to mitigate sample mixing and contact with the cap during transportation, but frozen transportation comes with additional cost and logistical challenges, leading many to prefer refrigerated transport. Therefore, it is critical to understand whether refrigerated shipping would affect measurement of these analytes. Our study showed that there was a less than 10% change in the concentration of Aβ42, p-Tau181, and t-Tau in CSF stored in low-bind polypropylene tubes when subjected to simulated refrigerated shipping conditions for 3 days, confirming the acceptability of refrigerated shipping.

Another important preanalytical factor in CSF testing is blood contamination. Blood can contaminate CSF due to a traumatic lumbar puncture and has been seen in approximately 15% of samples.^19^ Prior studies have shown that visible blood contamination can reduce Aβ concentrations.^20,21^ The hemolysis cutoff for Aβ42, p-Tau181, and t-Tau suggested by the manufacturer was 15 mg/dL (0.15 g/L), a concentration undetectable by eye. Although it is possible to measure the amount of free hemoglobin on another instrument, it is cumbersome and not always practical for every specimen. Therefore, we wanted to determine how sensitive these assays were to hemoglobin and establish a threshold of acceptability. We showed that up to at least 2.26 g/L free hemoglobin does not affect the results of p-Tau181 and t-Tau, but Aβ42 was affected by free hemoglobin in the sample, showing a more than 10% decrease in concentration at 2.26 g/L hemoglobin. Importantly, 0.9 g/L hemoglobin did not appreciably affect the measured Aβ42 concentration. At 0.9 g/L, blood contamination can be detected by eye, making identification of samples with blood contamination above the acceptable threshold easier. In addition, establishing a higher hemolysis threshold than the manufacturer’s claim will allow for reduced sample rejection, avoiding the need for sample re-collection.

The present study is not without limitations. Our sample size was small, limiting the statistical power and generalizability of the data. A more robust sample set would be useful to determine the reproducibility of this data. In addition, our study did not include freshly drawn CSF samples from patients but rather used residual specimens that had been frozen before our studies. Earlier work has shown differences in the degree of blood contamination interference in fresh vs frozen samples.^21^ It is possible that the interference we observed would be mitigated in fresh, never frozen CSF specimens. We also cannot rule out differences in fresh never frozen CSF stability compared with the stability we observed in residual frozen samples. A previous study, however, has suggested a low fresh-frozen effect on the stability of Aβ42, p-Tau181, and t-Tau.^22^ Finally, our transportation studies did not investigate varying sample volume in the tubes. All tubes used for our study were filled approximately 75% full. Cerebrospinal fluid fill volumes can affect Aβ42 recovery. Specifically, lower tube fill volumes have been associated with greater variability and poor recovery of Aβ42.^4^ Our data support refrigerated transportation of CSF low-bind tubes filled at least 75% full. Further investigation would be warranted to accept lower tube volumes.

This study demonstrated that refrigerated transportation, with sample mixing and inversion up to 3 days, does not affect the analytical measurement of Aβ42, p-Tau181, or t-Tau in CSF. Because frozen shipping is a barrier for some, this finding can widen the transport options, allowing both refrigerated and frozen transit. It also showed that hemolysis has a negligible effect on p-Tau181 and t-Tau measurement up to at least 2.26 g/L free hemoglobin. In contrast, measured Aβ42 was decreased with hemolysis at 2.26 g/L free hemoglobin but acceptable up to at least 0.9 g/L hemoglobin, which is more discernable by eye than the manufacturer’s suggested 0.15 g/L and will allow for simpler identification of unacceptable specimens.
